# Isolation, cultivation and molecular characterization of a new *Trypanosoma equiperdum* strain in Mongolia

**DOI:** 10.1186/s13071-016-1755-3

**Published:** 2016-08-31

**Authors:** Keisuke Suganuma, Sandagdorj Narantsatsral, Banzragch Battur, Shino Yamasaki, Davaajav Otgonsuren, Simon Peter Musinguzi, Batdorj Davaasuren, Badgar Battsetseg, Noboru Inoue

**Affiliations:** 1 National Research Center for Protozoan Diseases, OIE Reference Laboratory for Surra, Obihiro University of Agriculture and Veterinary Medicine, Inada, Obihiro, Hokkaido 080-8555 Japan; 2 Institute of Veterinary Medicine, Laboratory of Molecular Genetics, Mongolian University of Life Sciences, Zaisan, 17024 Ulaanbaatar Mongolia

**Keywords:** Dourine, In vitro culture, Maxicircle DNA, Mongolia, Soft agarose media, *Trypanosoma equiperdum*

## Abstract

**Background:**

*Trypanosoma equiperdum* causes dourine via sexual transmission in Equidae. *T. equiperdum* is classified under the subgenus *Trypanozoon* along with the *T. brucei* sspp. and *T. evansi*; however, the species classification of *Trypanozoon* remains a controversial topic due to the limited number of *T. equiperdum* reference strains*.* In addition, it is possible that some were misclassified *T. evansi* strains. Thus, there is a strong need for a new *T. equiperdum* strain directly isolated from the genital mucosa of a horse with a clinically- and parasitologically-confirmed dourine infection.

**Methods:**

Trypanosomes isolated from the urethral tract of a stallion with suspected dourine, were directly cultivated using soft agarose media at 37 °C in 5 % CO_2_. For molecular characterization, 18S ribosomal RNA (rRNA) gene, the internal transcribed spacer (ITS) and 8 maxicircle DNA regions were amplified by a PCR and their sequences were determined. To analyze the ratio of the kinetoplastic/akinetoplastic population, the kinetoplasts and the nuclei of trypanosomes were subjected to Hoechst staining and observed by fluorescence microscopy.

**Results:**

In addition to the clinical symptoms and the molecular diagnosis, this stallion was definitively diagnosed with dourine by the detection of trypanosomes in the urethral mucosa. These results strongly suggested that the isolated trypanosome was true *T. equiperdum. T. equiperdum* isolated from the urethral tract was adapted in vitro using soft agarose media. Based on the results of a phylogenetic analysis of 18S rRNA and ITS, this *T. equiperdum* isolate was classified into the *Trypanozoon* clade. In a PCR of the maxicircle DNA region, only NADH-dehydrogenase subunits 4 and 5 was amplified. Clear kinetoplasts were observed in most of the *T. equiperdum* isolates. In contrast, most culture-adapted *T. equiperdum* were of the akinetoplastic form.

**Conclusion:**

We concluded that our isolated trypanosome was the first confirmed case of *T. equiperdum* in Mongolia and named it “*T. equiperdum* IVM-t1”. *T. equiperdum* IVM-t1 was well adapted and propagated in soft agarose media, which indicates that this culture method is useful for isolation of *T. equiperdum* from horses with dourine.

**Electronic supplementary material:**

The online version of this article (doi:10.1186/s13071-016-1755-3) contains supplementary material, which is available to authorized users.

## Background

Dourine is caused by *Trypanosoma equiperdum* of the subgenus *Trypanozoon*. Unlike other trypanosomes, dourine is not transmitted by insect vectors; rather, it is transmitted by the infected horse via coitus. Thus, dourine has previously been distributed worldwide [1].

Subgenus *Trypanozoon* includes three subspecies of *T. brucei*. (*T. brucei brucei, T. b. gambiense* and *T. b. rhodesiense*), *T. evansi* and *T. equiperdum. T. brucei, T. evansi* and *T. equiperdum* have been classified based on their kinetoplast DNA (kDNA) components: *T. brucei* contains complete maxicircle kDNA, *T. evansi* completely lacks maxicircle kDNA, while the integrity of the maxicircle kDNA of *T. equiperdum* varies in each strain. Nevertheless, its classification within the subgenus *Trypanozoon* remains a controversial topic because it has been hypothesized that a very close evolutionary relationship exists among the trypanosome species of *Trypanozoon* [2, 3]. Moreover, many of *T. equiperdum* strains were isolated over 50 years ago, and it was hypothesized that some of the isolates were misclassified *T. evansi* strains [3]. Some of the new *T. equiperdum* strains were recently isolated in Italy and Ethiopia from horses with suspected dourine infections [4, 5]. However, these *T. equiperdum* strains were not directly isolated from the genital mucosa (the primary site of infectious lesions of *T. equiperdum*). Instead, they were isolated from udder secretion samples or jugular venous blood. Thus, new *T. equiperdum* strains that are directly isolated from the infectious lesions of horses with clinically- and parasitologically-confirmed dourine have long been needed for further studies on *T. equiperdum* and dourine.

Previous reports have shown the prevalence of equine trypanosomosis in Mongolia and Kazakhstan to be 6–8 % and 16.8 %, respectively [6, 7]. However, these reports did not identify the causative species because it is very difficult to distinguish *T. equiperdum* from *T. evansi* using serological diagnostic techniques. On the other hand, the re-emergence of dourine was reported in Italy in May 2011 when the characteristic symptoms of dourine (e.g. paralysis of the lip, edema of the vulva and cutaneous wheals) were observed in a number of horses [8]. A serological test revealed that approximately 0.5 % of the equine population was diagnosed as dourine-positive by CFT, moreover, dourine positivity (based on the case definition) has been confirmed in 0.03 % of all horses [9]. Moreover, trypanosomes were observed in the skin lesions and were isolated from infected horses by intrascrotal inoculation [5].

Our ongoing epidemiological research suggests high prevalence of trypanosomoses in horses in Mongolia. In the present study, dourine was diagnosed parasitologically via the detection of *T. equiperdum* in the urethral tract of a stallion with the characteristic clinical symptoms. Moreover, we established a new true *T. equiperdum* strain that was isolated from the urethral tract of a dourine-infected stallion. Furthermore, we used a PCR to molecularly characterize this new strain of *T. equiperdum*.

## Methods

### The identification of the stallion with suspected dourine

**Fig. 1:**
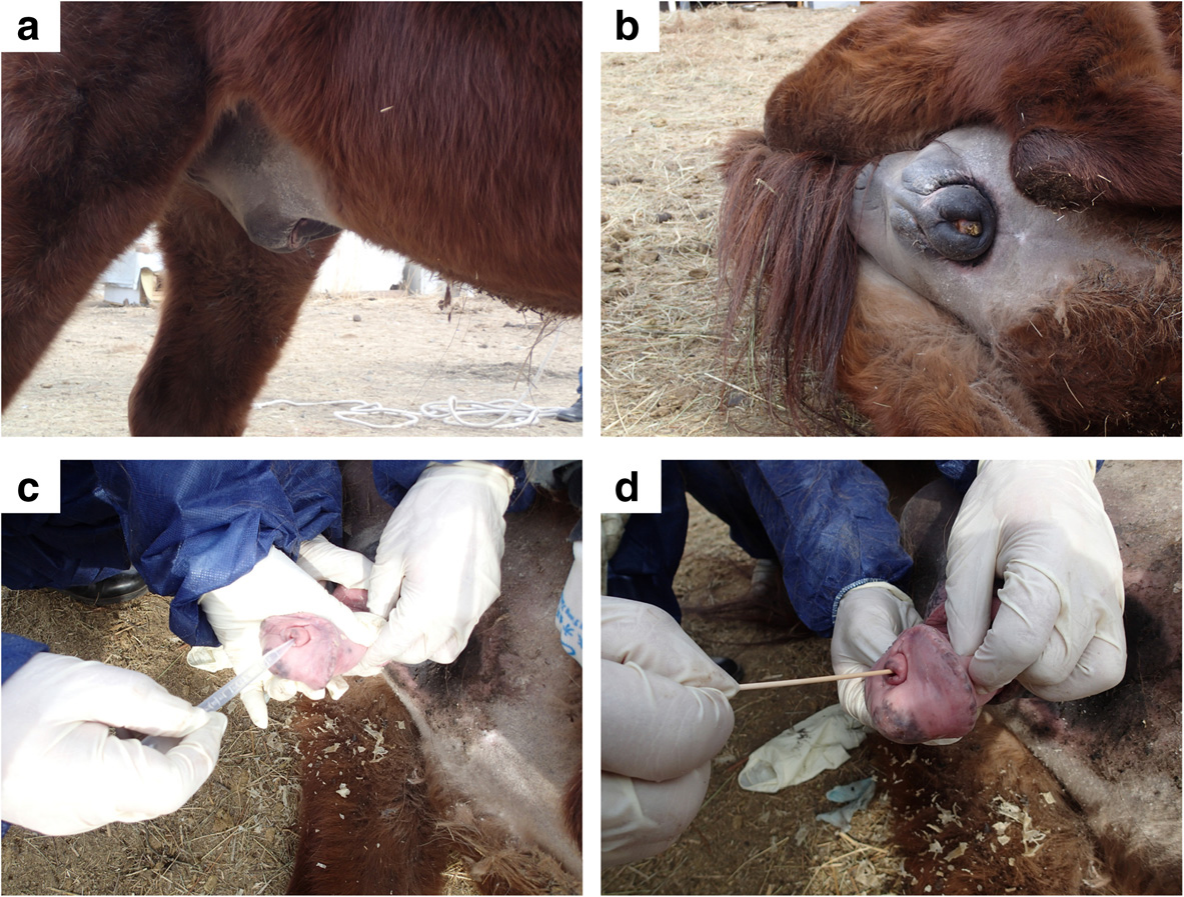
The swelling of the genital organ of the dourine-infected stallion and sampling of trypanosomes from the urethral tract. **a** and **b** The swelling of the genital organ of the dourine-infected stallion. **c** HMI-9 was injected into the urethral tract using a transfer pipette to detach adherent *T. equiperdum* from the urethral tract mucosa. **d** Sampling of *T. equiperdum* from the urethral tract mucosa using a cotton swab

The brown-colored 7-year-old stallion was bred in an equestrian farm in Töv aimag in Mongolia. It was suspected of being infected with dourine based on the presence of slight paraphimosis and edema in the genital organ (Fig. 1a and b). The blood parameters (white blood cell [WBC], red blood cell [RBC], hemoglobin [HGB], hematocrit [HCT], mean corpuscular volume [MCV], mean corpuscular hematocrit [MCH], mean corpuscular hemoglobin concentration [MCHC], and platelet [PLT] count) were measured using a Celltac α (Nihon Khoden, Tokyo, Japan); while the measurement of the blood chemistry parameters (alkaline phosphatase [ALP], alanine transaminase [ALT], aspartate aminotransferase [AST], albumin, total protein, total cholesterol, bilirubin, blood urea nitrogen [BUN], creatinine, and amylase) was outsourced (Table 1). Molecular diagnoses were then performed using DNA extracted from the blood and serum of the stallion by a KIN-PCR, a complement fixation test (CFT), a recombinant *T. evansi* GM6-4r antigen-based immunochromatographic test (ICT) and an enzyme-linked immunosorbent assay (ELISA) [10–13]. The collected cerebrospinal fluid (CSF) was centrifuged to concentrate the trypanosomes. Trypanosomes in the sediment of the CSF and in the blood were observed by the wet blood film method. Parasitemia in the blood was estimated using Herbert & Lumsden’s method [14].

**Table 1:** The clinical symptoms, blood parameters and blood chemistry of the dourine-infected stallion

Clinical symptoms	Slight edema in the genital organ
	Slight paraphimosis
	A large amount of smegma around the penis
	Small skin lesion
	Slight anemia
Blood parameters	WBC: 12.9 ×10^3^ cells/μl (slightly high)
	RBC: 6.32 ×10^6^ cells/μl (slightly low)
	HGB: 10.6 g/dl (slightly low)
	HCT: 30.9 % (slightly low)
	Other parameters: normal (MCV, MCH, MCHC, PLT)
Blood chemistry parameters	Normal (ALP, ALT, AST, albumin, total protein, total cholesterol, bilirubin, BUN, creatinine, amylase)
Parasitemia	Swab of the urethral tract mucosa: a lot of trypanosomes
	Blood: < 2.5 × 10^5^ cells/ml^a^ (1 trypanosome per 500 fields; magnification 400×)
	Cerebrospinal fluid: relatively a lot of trypanosomes in sediment

^a^Parasitemia was estimated using Herbert & Lumsden's method [14]

### Isolation and cultivation of *T*. *equiperdum* in vitro

To isolate *T. equiperdum* from the urethral tract of the stallion, Hirumi’s modified Iscove’s medium-9 supplemented with 20 % heat-inactivated adult horse serum (HMI-9) [15] was injected into the urethral tract of the stallion with a suspected dourine infection to detach *T. equiperdum* from the urethral tract mucosa (Fig. 1c). Subsequently, *T. equiperdum* was sampled from the urethral tract mucosa using a cotton swab (Fig. 1d). The *T. equiperdum* specimens from the urethral tract mucosa were smeared on slide glasses, fixed with 100 % methanol and stained with Giemsa for microscopic observation. The major axis of isolated *T. equiperdum* was measured using a Nikon Eclipse Ci microscope and the Nice D software program (Nikon Corporation, Tokyo, Japan).

The *T. equiperdum* isolates from the urethral tract were centrifuged with HMI-9 media at 3000× *g* for 10 min at room temperature. The pellets containing *T. equiperdum* and urethral mucosa cells were washed twice with HMI-9. Finally, *T. equiperdum* suspended in HMI-9 was spread on soft agarose media (HMI-9 with 0.8 % low gelling agarose [Type VII, Sigma-Aldrich Japan, Tokyo, Japan]) at 37 °C in 5 % CO_2_. Culture-adapted *T. equiperdum* specimens were cryopreserved in horse serum supplemented with 10 % dimethyl sulfoxide at -80 °C.

### DNA extraction

Total DNA of *T. equiperdum* was extracted and purified using TE-saturated phenol (Sigma-Aldrich, Japan) and phenol-chloroform-isoamyl alcohol solution (Sigma-Aldrich, Japan) [16]. Purified DNA was kept at -30 °C until use.

### PCR amplification and phylogenetic analysis of 18S rRNA gene and ITS

The 18S ribosomal RNA (18S rRNA) gene and the internal transcribed spacer (ITS) region of the Mongolian isolate and the reference strains of *T. equiperdum* (the total DNA of the STIB818, STIB841, STIB842 and BoTat1.1 strains were all kindly provided by Dr. Zhao-Rong Lun of Sun Yat-Sen University in the People’s Republic of China) were amplified and cloned into the pCR 2.1 cloning vector (Thermo Fisher Scientific K.K., Tokyo, Japan), and the sequences were analyzed using an ABI3100 genomic analyzer (Thermo Fisher Scientific K.K.) [17]. In addition to these *T. equiperdum* sequence data, the reference sequences from the NCBI database (*T. brucei* [Accession No. AC012647.18], *T. b. rhodesiense* [AJ009142], *T. b. gambiense* [FN554966.1 and AJ009141] and *T. evansi* [AB551922.1, AY912277, AY912279 and D89527.1]) were included in the phylogenetic analyses. The phylogenetic analyses of the 18S rDNA and ITS regions were performed using the neighbor-joining method using the MEGA 7 software program.

### Maxicircle kDNA characterization

The target loci on the maxicircle genes were amplified from the total DNA of the Mongolian *T. equiperdum* isolate. In addition, the total DNA from *T. b. brucei* GUTat3.1, *T. evansi* IL3960 and *T. equiperdum* STIB818, STIB841, STIB842 and BoTat1.1 were used for maxicircle gene amplification in order to compare the maxicircle kinetoplast DNA (kDNA) sequences among the trypanosome species. The primer sequences for each of the target loci on the maxicircle gene have been described previously (Additional file 1: Table S1) [18]. The amplicons were cloned into pCR 2.1 cloning vector (Thermo Fisher Scientific K.K.), and the sequences were analyzed using an ABI3100 genomic analyzer (Thermo Fisher Scientific K.K.).

### The measurement of kinetoplastic and akinetoplastic* T. equiperdum* population

Culture-adapted *T. equiperdum* and *T. equiperdum* that were directly obtained from the urethral tract and CSF of an infected stallion were spread over glass slides printed with highly water-repellent marking (Matsunami Glass Ind., Ltd., Tokyo, Japan), air-dried, and fixed with 100 % methanol for 10 min at room temperature. The specimens were blocked with 5 % skim milk in TBS supplemented with 0.05 % tween for 1 h at room temperature. The specimens were then incubated with a primary antibody (anti-recombinant *T. congolense* α-tubulin serum) [19]. Next, the slides were incubated with a secondary antibody (Alexa Fluor 488 goat anti-rabbit IgG [H + L], Thermo Fisher Scientific K.K.) with Hoechst 33342 (Dojindo, Co. Ltd., Kumamoto, Japan). The specimens were observed by confocal laser scanning microscopy (Leica TCS SP5, Leica Microsystems, Wetzlar, Germany).

## Results

### *T*. *equiperdum* isolated from the urethral tract of the dourine-infected stallion

**Fig. 2:**
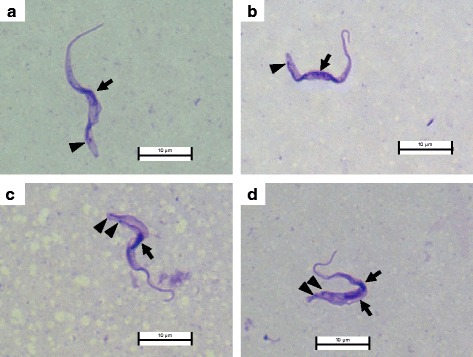
Giemsa-stained *T. equiperdum* isolated from the urethral tract of the dourine-infected stallion. **a** and **b** (1K1N) show the single form of *T. equiperdum*. **c** (2K1N) and **d** (2K2N) show the dividing form of *T. equiperdum*. Arrow: nucleus; arrowhead: kinetoplast

Dourine was suspected based on the swelling of the genital organ, slight paraphimosis, a large amount of smegma and a small skin lesion on the stallion (Fig. 1a and b). In addition, slight anemia was suggested based on a hematological examination (Table 1). Moreover, the CFT, ICT and ELISA results strongly suggested a trypanosome infection (data not shown). However, these molecular diagnostic methods were not capable of differentiating between *T. equiperdum* and *T. evansi*. Thus, we could not eliminate the possibility of a *T. evansi* infection (Surra) based on these results. In addition to suspecting dourine based on the clinical symptoms and the molecular diagnostic results, actively moving trypanosomes were detected from the mucosa of the urethral tract by microscopy. Moreover, the parasitemia in the blood was very low, while that in the sediment of the CSF was relatively high (Table 1). We therefore concluded that this stallion was infected with *T. equiperdum*. The microscopic observation of Giemsa-stained trypanosomes was used for the morphological characterization of the *T. equiperdum* isolate of the naturally infected stallion. Kinetoplasts were clearly stained and observed in all of the trypanosomes. Single-form and dividing-form (2K1N and 2K2N) trypanosomes with a free flagellum were observed (Fig. 2). The major axis of the trypanosomes was 26.4 ± 3.1 μm (mean ± standard deviation, *n* = 27).

### Culture adaptation of the *T*. *equiperdum* isolate

*T. equiperdum* isolated from urethral tract of the dourine-infected stallion was directly transferred into an in vitro culture. They were adapted in a soft agarose media culture system. Unlike the culturing of other bloodstream-form trypanosomes, the primary culture of isolated *T. equiperdum* could not be successfully propagated in HMI-9 liquid media. The parasites were found to be attached and actively moving on the surface of soft agarose media (Additional file 2: Movie 1). Some of them invaded the soft agarose and propagate under the surface of soft agarose during cultivation. Unlike *T. brucei*, they could not form colonies on the surface of soft agar [20]. This new culture-adapted *T. equiperdum* strain was named, “IVM-t1 (*T. equiperdum* isolated in the Institute of Veterinary Medicine from Töv aimag dourine horse no. 1), 2015.”

### Phylogenetic analyses

The phylogenetic relationships were inferred from a comparison of the 18S rRNA and ITS sequences of *T. equiperdum* and other *Trypanozoon*. Like the other *T. equiperdum* strains, the newly isolated *T. equiperdum* IVM-t1 strain belonged to the *Trypanozoon* clade (Additional file 3: Figure S1).

### *T*. *equiperdum* IVM-t1 strain lacks maxicircle integrity

**Fig. 3:**
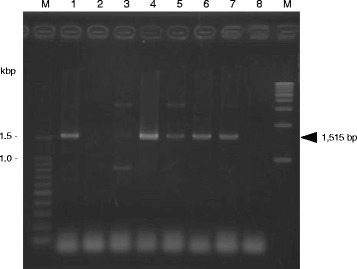
The PCR to detect NADH-dehydrogenase subunits 4 and 5 (ND4-ND5). A gel electrophoresis image of the PCR products of NADH-dehydrogenase subunits 4 and 5 (ND4-ND5). The arrowhead at 1515 bp indicates the amplicon of ND4-ND5 in lanes 1 and 3 to 7. M: 100 bp and 1 kbp DNA ladders, Lanes 1 to 8 are *T. b. brucei* GUTat3.1, *T. evansi* IL3960, *T. equiperdum* IVM-t1, STIB818, STIB841, STIB842, BoTat1.1 strains and a negative control (distilled water), respectively

Eight PCRs targeting the maxicircle genes were performed to compare the maxicircle integrity among the *Trypanozoon* parasites. The maxicircle PCR of *T. evansi* IL3960, *T. brucei* GUTat3.1 and *T. equiperdum* STIB818, STIB841, STIB842 and BoTat1.1 strains (as the reference strains) showed the same results as previous reports (Table 2) [18]. On the other hand, only NADH-dehydrogenase subunits 4 and 5 (ND4-ND5) was amplified in *T. equiperdum* IVM-t1 strain (Table 2, Fig. 3 and Additional file 3: Figure S2). The PCR signal of ND4-ND5 in the *T. equiperdum* IVM-t1 strain was somewhat weaker than the ND4-ND5 signals of the other trypanosomes. Sequence analyses showed that the other bright PCR bands (e.g. around the 1.5 kbp band in lane 3 of Additional file 3: Figure S2-A) were not the maxicircle target genes.

**Table 2:** Summary of the maxicircle PCR results

Target locus^a^	*T. b. brucei*	*T. evansi*	*T. equiperdum*				
	GUTat3.1	IL3960	IVM-t1	STIB818	STIB841	STIB842	BoTat1.1
NAD7	P	N	N	N	P	P	P
Cox2	P	N	N	N	P	P	P
A6	P	N	N	N	P	P	P
12S rRNA	P	N	N	P	P	P	P
ND7-CyB	P	N	N	N	P	P	P
MURF1-ND1	P	N	N	N	P	P	P
MURF2-Cox1	P	N	N	N	P	P	P
ND4-ND5	P	N	P	P	P	P	P

All of the PCR product sequences were confirmed by a sequence analysis and the NCBI BlastN software program

*Abbreviations*: *P* Positive, *N* Negative

^a^NAD7, NADH-dehydrogenase subunit 7; Cox2, Cytochrome oxidase subunit 2; A6, ATPase subunit 6; 12S rRNA, 12S ribosomal RNA; ND7-CyB, NADH-dehydrogenase subunit 7-cytochrome b; MURF-ND1, Maxicircle unknown reading frame-NADH dehydrogenase subunit 1; MURF2-Cox1, Maxicircle unknown reading frame 2-cytochrome oxidase subunit 1 and ND4-ND5, NADH-dehydrogenase subunits 4–5

### The majority of the culture-adapted *T*. *equiperdum* IVM-t1 strain population was akinetoplastic after long-term in vitro cultivation

**Fig. 4:**
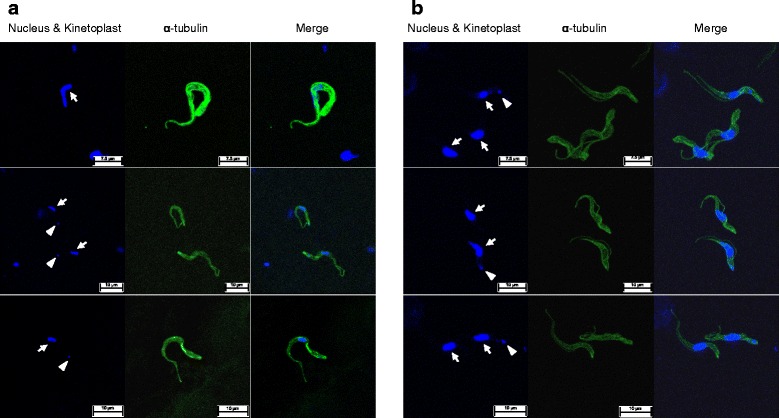
Akinetoplastic and kinetoplastic *T. equiperdum* in wild-type and in vitro culture. **a** *T. equiperdum* isolated from the urethral tract. Upper panel, akinetoplastic form; middle and lower panels, kinetoplastic form. **b** Kinetoplastic and akinetoplastic *T. equiperdum* in vitro culture. Arrow: nucleus; arrowhead: kinetoplast

Kinetoplasts were clearly observed in almost all of the *T. equiperdum* specimens from the urethral tract by Giemsa and DNA staining (1 out of 160 [0.63 %] trypanosomes were akinetoplastic) (Figs. 2 and 4a). In addition, akinetoplastic *T. equiperdum* accounted for a minor part of the trypanosome population in the CSF (6 out of 326 [1.84 %] of the trypanosomes were akinetoplastic). On the other hand, kinetoplasts were not observed in most of the culture-adapted from *T. equiperdum* IVM-t1 strain (480 out of 500 [96.0 %] trypanosomes were akinetoplastic) (Fig. 4b).

## Discussion

*T. equiperdum* is a cosmopolitan trypanosome that causes dourine via sexual transmission in the Equidae. The phylogenetic relationships of the subgenus *Trypanozoon* (*T. brucei* sspp.*, T. evansi* and *T. equiperdum*) have been unclear, and serological methods for differentially diagnosing the various *Trypanozoon* trypanosomes have not been established due to the lack of information about the *T. equiperdum* genome. Many of the *T. equiperdum* strains were isolated more than 50 years ago [3]. Recently new *T. equiperdum* strains were isolated from horses with suspected dourine in Italy and Ethiopia [4, 5]. However, these *T. equiperdum* strains were not directly isolated from the genital mucosa (infectious lesion of *T. equiperdum*). In the present study, we isolated *T. equiperdum* from the urethral tract of a dourine-infected stallion and established a new culture-adapted *T. equiperdum* strain. Moreover, we conducted the molecular characterization of this *T. equiperdum* strain based on the 18S rRNA, ITS and maxicircle gene sequences.

In addition to the clinical symptoms that led us to suspect dourine and the results of the molecular diagnosis, live trypanosomes were observed on a wet smear from the urethral tract mucosa of one stallion in Mongolia (Table 1). Moreover, trypanosomes were detected in the blood (< 2.5 × 10^5^ cells/ml) and CSF (relatively high parasitemia was observed in the sediment) (Table 1). Although *T. equiperdum* mainly parasitizes in the tissue, it is rarely observed in the bloodstream of horses with chronic infection [1]. We therefore concluded that this stallion was infected with *T. equiperdum*. Since the dividing-form and actively moving trypanosomes were observed, it was concluded that *T. equiperdum* parasites were propagating in the urethral tract mucosa and that they caused dourine in this stallion (Fig. 2). Our ongoing surveillance project in Mongolia also revealed Surra epidemics in other domestic animals (data not shown). Previous reports showed that *Tabanus* spp. were trapped in Hustai National Park, Mongolia [21]. These results indicated that the *Tabanus* spp. that can transmit *T. evansi* can be found in Mongolia. Although the parasitemia was very low, *T. equiperdum* parasitized the bloodstream in this infected stallion. We therefore could not completely exclude the possibility of the mechanical transmission of *T. equiperdum* due to blood sacking by the *Tabanus* spp.

Isolated *T. equiperdum* parasites were adapted and proliferated well using HMI-9 soft agarose media as a primary trypanosome culture (Additional file 2: Movie 1). While isolated, *T. equiperdum* could not be propagated in HMI-9 liquid media in primary trypanosome cultures. Furthermore, unlike *T. brucei*, to proliferate on the surface of the soft agarose media, *T. equiperdum* invaded the surface of soft agarose media but could not form colonies [20]. After the infection of the genital mucosa via coitus, *T. equiperdum* invade the tissue and parasitize in the blood, lymph, CSF and sub-cutaneous lesions [5]. These results suggest that the soft agarose media mimics the natural environment of the host genital mucosa. Thus, the isolated *T. equiperdum* were well adapted and proliferated using soft agarose media but did not adapt in liquid media. This culture system will be useful for the future isolation of *T. equiperdum* from dourine-infected horses in the field.

A phylogenetic analysis using 18S rRNA and the ITS region revealed that, along with the other *T. equiperdum* strains, the *T. equiperdum* IVM-t1 strain was a member of the *Trypanozoon* clade (Additional file 3: Figure S1). *T. equiperdum* and *T. evansi* were hypothesized to have independently evolved from ancestral *T. brucei* at least four times [22, 23]. Thus, *T. equiperdum* is a polyphyletic group and, based on the results of a previous gnomic analysis, is considered to be a subspecies of *T. brucei*. However, Desquesnes et al. [24] suggested that *T. equiperdum* and *T. evansi* should keep their current species status based on the significant biological and parasitical differences between these species. A RoTat 1.2 VSG PCR, which was performed to analyze the sequence of the amplicon, was negative (data not shown). Moreover, only NADH-dehydrogenase subunit 4 and 5 (ND4-ND5) was amplified in the maxicircle of this strain of *T. equiperdum* (Table 2 and Fig. 3). The majority of the culture-adapted *T. equiperdum* IVM-t1 strain population was composed of akinetoplastic-form parasites; thus, the weak ND4-ND5 signal was caused by the small amount of maxicircle template DNA because of the small population in kinetoplastic culture-adapted *T. equiperdum* IVM-t1. The results of this molecular analysis also supported that this isolated trypanosome was *T. equiperdum*.

Many of the akinetoplastic *Trypanozoon* trypanosome strains have been established from a parental kinetoplastic strain by the supplementation of DNA binding drugs or RNA interference during in vitro culture [25]. The predominance of the akinetoplastic *T. evansi* and *T. equiperdum* population was also induced from a kinetoplastic parental trypanosome by long-term aseptic cultivation [26]. In the present study, the *T. equiperdum* IVM-t1 strain was cultivated without any drug supplementation based on the intention to remove kinetoplasts from the kinetoplastic parental *T. equiperdum* over a one-year cultivation period. However, the majority of the population (96.0 %) in culture-adapted *T. equiperdum* IVM-t1 strain was akinetoplastic-form, despite akinetoplastic *T. equiperdum* being a minority population in the parental *T. equiperdum* population (0.63 %) (Fig. 4). We cannot conclude that the predominance of akinetoplastic forms in the culture-adapted *T. equiperdum* IVM-t1 strain were simply selected from parental akinetoplastic *T. equiperdum*, which was the minority population in the urethral tract, or induced rapid kinetoplast loss from the parental kinetoplastic *T. equiperdum* during long-term in vitro cultivation without any drug supplementation. The rapid loss of kinetoplasts in culture-adapted *T. equiperdum* IVM-t1 strain might be due to the acquisition of a rapid proliferative potential in the culture, similarly to the proliferation of unregulated cancer cells in the host [27]. *T. equiperdum* is usually distinguished from *T. evansi* by an analysis of the kDNA (by a PCR); however, it was not possible to detect dyskinetoplastic *T. equiperdum* strains (such as the culture-adapted *T. equiperdum* IVM-t1 strain) by an analysis of the kDNA. Other species-specific markers are therefore expected to be useful for the definitive diagnosis of *Trypanozoon* infections. We performed a further comparative whole genomic analysis and transcriptome analysis using a next generation sequencing technique to compare the *T. equiperdum* IVM-t1 strain with other *Trypanozoon* species. Based on these results, we intend to develop *T. equiperdum*-specific PCR methods and serodiagnostic methods that can be used to definitively diagnose *Trypanozoon* infections and reveal the evolution, origin and pathogenic effects of *Trypanozoon*.

## Conclusions

In conclusion, we successfully isolated *T. equiperdum* from the urethral tract mucosa of a dourine-infected stallion with characteristic clinical symptoms using soft agarose media. We therefore propose that this *T. equiperdum* IVM-t1 strain is a new *T. equiperdum* reference strain. Whole genome and transcriptome analyses using this new reference *T. equiperdum* strain are expected to reveal the phylogenetic relationship between *Trypanozoon* and to be useful in the development of novel methods for diagnosing dourine.

## Additional files

Additional file 1: Table S1. The PCR primers used in the present study. (DOCX 25 kb) Additional file 2:
**Movie 1.** Culture-adapted *T*. *equiperdum* IVM-t1 were proliferated on the surface of soft agarose media. (M4V 8770KB) Additional file 3: Figure S1. The phylogenetic tree of the 18S rRNA and ITS region. A phylogenetic analysis was performed using the *T. equiperdum* IVM-t1, SITB818, STIB841, STIB842, BoTat1.1, *T. evansi* Tansui (Accession No. D89527.1), Cairo (AB551922.1), KAI.2 (AY912277), Sam.2 (AY912279.1), *T. brucei* TREU927 (AC012647), *T. b. gambiense* DAL972 (FN554966.1), *T. b. gambiense* Tsuua (AJ009141) and *T. b. rhodesiense* Utro (AJ009142) sequences. A: A phylogenetic tree based on the 18S rRNA sequence. B: A phylogenetic tree based on the ITS sequence. **Figure S2.** The maxicircle PCR of the *Trypanozoon* species. Gel electrophoresis images of the PCR products are shown in A to G, NADH-dehydrogenase subunit 7 (NAD7; 383 bp), Cytochrome oxidase subunit 2 (Cox2; 1747 bp), ATOas subunit 6 (A6; 299 bp), 12S ribosomal RNA (12S rRNA; 1597 bp in *T. b. brucei* GUTat3.1 strain and *T. equiperdum* STIB818 strain, 1415 bp in *T. equiperdum* STIB841, STIB842, BoTat1.1 strains, respectively), NADH-dehydrogenase subunit 7-cytochromeB (ND7-CyB; 1450 bp), Maxicircle unknown reading frame-NADH dehydrogenase subunit 1 (MURF-ND1; 1779 bp) and Maxicircle unknown reading frame 2-cytochrome oxidase subunit 1 (MURF2-Cox1; 1551 bp), respectively. M: the 100 bp and 1 kbp DNA ladders; Lanes 1 to 8 show *T. b. brucei* GUTat3.1, *T. evansi* IL3960, *T. equiperdum* IVM-t1, STIB818, STIB841, STIB842, BoTat1.1 strains and negative control (distilled water), respectively. (PPTX 164 kb)

## Abbreviations

18S rRNA: 18S ribosomal RNA
ALP: Alkaline phosphatase
ALT: Alanine transaminase
AST: Aspartate aminotransferase
BUN: Blood urea nitrogen
CFT: Complement fixation test
CSF: Cerebrospinal fluid
ELISA: Enzyme-linked immunosorbent assay
HCT: Hematocrit
HGB: Hemoglobin
HMI-9: Hirumi’s modified Isocove’s medium-9
ICT: Immunochromatographic test
ITS: Internal transcribed spacer
kDNA: Kinetoplast DNA
MCH: Mean corpuscular hematocrit
MCHC: Mean corpuscular hemoglobin concentration
MCV: Mean corpuscular volume
ND4-ND5: NADH-dehydrogenase subunits 4 and 5
PLT: Platelet
RBC: Red blood cell
WBC: White blood cell

## References

[1] Brun R, Hecker H, Lun ZR (1998). *Trypanosoma evansi* and *T. equiperdum*: distribution, biology, treatment and phylogenetic relationship (a review). Vet Parasitol.

[2] Li FJ, Lai DH, Lukes J, Chen XG, Lun ZR (2006). Doubts about *Trypanosoma equiperdum* strains classed as *Trypanosoma brucei* or *Trypanosoma evansi*. Trends Parasitol.

[3] Claes F, Büscher P, Touratier L, Goddeeris BM (2005). *Trypanosoma equiperdum*: master of disguise or historical mistake?. Trends Parasitol.

[4] Hagos A, Goddeeris BM, Yilkal K, Alemu T, Fikru R, Yacob HT, Feseha G, Claes F (2010). Efficacy of Cymelarsan (R) and Diminasan (R) against *Trypanosoma equiperdum* infections in mice and horses. Vet Parasitol.

[5] Pascucci I, Di Provvido A, Camma C, Di Francesco G, Calistri P, Tittarelli M, Ferri N, Scacchia M, Caporale V (2013). Diagnosis of dourine in outbreaks in Italy. Vet Parasitol.

[6] Clausen PH, Chuluun S, Sodnomdarjaa R, Greiner M, Noeckler K, Staak C, Zessin KH, Schein E (2003). A field study to estimate the prevalence of *Trypanosoma equiperdum* in Mongolian horses. Vet Parasitol.

[7] Claes F, Ilgekbayeva GD, Verloo D, Saidouldin TS, Geerts S, Buscher P, Goddeeris BM (2005). Comparison of serological tests for equine trypanosomosis in naturally infected horses from Kazakhstan. Vet Parasitol.

[8] Vulpiani MP, Carvelli A, Giansante D, Iannino F, Paganico D, Ferri N (2013). Reemergence of dourine in Italy: clinical cases in some positive horses. J Equine Vet Sci.

[9] Calistri P, Narcisi V, Atzeni M, De Massis F, Tittarelli M, Mercante MT, Ruggieri E, Scacchia M (2013). Dourine reemergence in Italy. J Equine Vet Sci.

[10] Nguyen TT, Zhou M, Ruttayaporn N, Nguyen QD, Nguyen VK, Goto Y, Suzuki Y, Kawazu S, Inoue N (2014). Diagnostic value of the recombinant tandem repeat antigen TeGM6-4r for surra in water buffaloes. Vet Parasitol.

[11] Nguyen TT, Ruttayaporn N, Goto Y, Kawazu S, Sakurai T, Inoue N (2015). A TeGM6-4r antigen-based immunochromatographic test (ICT) for animal trypanosomosis. Parasitol Res.

[12] Desquesnes M, McLaughlin G, Zoungrana A, Davila AM (2001). Detection and identification of *Trypanosoma* of African livestock through a single PCR based on internal transcribed spacer 1 of rDNA. Int J Parasitol.

[13] Thuy N, Goto Y, Lun ZR, Kawazu SI, Inoue N (2012). Tandem repeat protein as potential diagnostic antigen for *Trypanosoma evansi* infection. Parasitol Res.

[14] Herbert WJ, Lumsden WH (1976). *Trypanosoma brucei*: a rapid “matching” method for estimating the host’s parasitemia. Exp Parasitol.

[15] Hirumi H, Hirumi K (1991). In vitro cultivation of *Trypanosoma congolense* bloodstream forms in the absence of feeder cell layers. Parasitology.

[16] Sambrook J, Russell DW, Sambrook J (2006). The condensed protocols from molecular cloning: a laboratory manual.

[17] Da Silva FM, Noyes H, Campaner M, Junqueira ACV, Coura JR, Añez N, Shaw JJ, Stevens JR, Teixeira MMG (2004). Phylogeny, taxonomy and grouping of *Trypanosoma rangeli* isolates from man, triatomines and sylvatic mammals from widespread geographical origin based on SSU and ITS ribosomal sequences. Parasitology.

[18] Lai DH, Hashimi H, Lun ZR, Ayala FJ, Lukes J (2008). Adaptations of Trypanosoma brucei to gradual loss of kinetoplast DNA: *Trypanosoma equiperdum* and *Trypanosoma evansi* are petite mutants of *T. brucei*. Proc Natl Acad Sci U S A.

[19] Suganuma K, Sarwono AE, Mitsuhashi S, Jakalski M, Okada T, Nthatisi M, Yamagishi J, Ubukata M, Inoue N (2016). Mycophenolic acid and its derivatives as potential chemotherapeutic agents targeting inosine monophosphate dehydrogenase in *Trypanosoma congolense*. Antimicrob Agents Chemother.

[20] Carruthers VB, Cross GAM (1992). High-efficiency clonal growth of blood-stream-form and insect-form *Trypanosoma brucei* on agarose plates. Proc Natl Acad Sci U S A.

[21] King SRB, Gurnell J (2010). Effects of fly disturbance on the behaviour of a population of reintroduced Przewalski horses (*Equus ferus przewalskii*) in Mongolia. Appl Anim Behav Sci.

[22] Carnes J, Anupama A, Balmer O, Jackson A, Lewis M, Brown R, Cestari I, Desquesnes M, Gendrin C, Hertz-Fowler C (2015). Genome and phylogenetic analyses of *Trypanosoma evansi* reveal extensive similarity to *T. brucei* and multiple independent origins for dyskinetoplasty. PLoS Negl Trop Dis.

[23] Sanchez E, Perrone T, Recchimuzzi G, Cardozo I, Biteau N, Aso PM, Mijares A, Baltz T, Berthier D, Balzano-Nogueira L (2015). Molecular characterization and classification of *Trypanosoma* spp. Venezuelan isolates based on microsatellite markers and kinetoplast maxicircle genes. Parasit Vectors.

[24] Desquesnes M, Holzmuller P, Lai DH, Dargantes A, Lun ZR, Jittaplapong S (2013). *Trypanosoma evansi* and surra: a review and perspectives on origin, history, distribution, taxonomy, morphology, hosts, and pathogenic effects. Biomed Res Int.

[25] Schnaufer A, Domingo GJ, Stuart K (2002). Natural and induced dyskinetoplastic trypanosomatids: how to live without mitochondrial DNA. Int J Parasitol.

[26] Kaminsky R, Schmid C, Lun ZR (1997). Susceptibility of dyskinetoplastic *Trypanosoma evansi* and *T. equiperdum* to isometamidium chloride. Parasitol Res.

[27] Lun ZR, Lai DH, Wen YZ, Zheng LL, Shen JL, Yang TB, Zhou WL, Qu LH, Hide G, Ayala FJ. Cancer in the parasitic protozoans *Trypanosoma brucei* and *Toxoplasma gondii*. Proc Natl Acad Sci USA. 2015;112(29):8835–42. 10.1073/pnas.1502599112 PMC4517281 26195778

