# Supplementary material for: Isolation, cultivation and molecular characterization of a new Trypanosoma equiperdum strain in Mongolia
Source: Parasit Vectors. 2016 Aug 31;9(1):481. doi: 10.1186/s13071-016-1755-3 (PMC5007690; doi:10.1186/s13071-016-1755-3)
Supplement: Additional file 1: Table S1. — The PCR primers used in the present study. (DOCX 25 kb) [file 13071_2016_1755_MOESM1_ESM.docx]

**Supplementary Table 1. The PCR primers used in the present study**

| Target locus^a^ |  | Sequence | Expected size | Reference |
| --- | --- | --- | --- | --- |
| ND7 | Forward | 5’-AGA CAG ACG ACA GTG TCC-3’ | 383 bp ^b^ | Lai *et al.*, 2008 (18) |
|  | Reverse | 5’-AAG CCG CTA TCG ATT CC-3’ |  |  |
| Cox2 | Forward | 5’-TCC ATC AGT AAT AGG AGT AAG AAG-3’ | 1,747 bp ^b^ |  |
|  | Reverse | 5’-CTT TTA AAA AAA AAC TAA AAA ATG ATA T-3’ |  |  |
| A6 | Forward | 5’-AGG AAT TTT GGG CGG AAG A-3’ | 299 bp ^b^ |  |
|  | Reverse | 5’-CCC TAA CCT TTC CTG CTC-3’ |  |  |
| 12S rRNA | Forward | 5’-GGT TTT AGC TAT TTT AAA TTC CAA C-3’ | 1,597 bp ^b^ |  |
|  | Reverse | 5’-GCT ACA ATA ATA TTG CAA TTG AGG-3’ |  |  |
| ND7-CyB | Forward | 5’-AAG GGA GCA GAT TCG AAC G-3’ | 1,450 bp ^b^ |  |
|  | Reverse | 5’-CCA ATT TAT AAA TAT AAC ATA CAT CAT AC-3’ |  |  |
| MURF1-ND1 | Forward | 5’-CAC AAA TAG TAA ACT AAC GAT AGC-3’ | 1,779 bp ^b^ |  |
|  | Reverse | 5’-CGT TTT ATC TGT CTT ATG TGG GTA-3’ |  |  |
| MURF2-Cox1 | Forward | 5’-AGT AGA AGT TGT TAT ATA TTG ATG CC-3’ | 1,551 bp ^b^ |  |
|  | Reverse | 5’-TAA GTG GGT TTT TGA CTG AAG AG-3’ |  |  |
| ND4-ND5 | Forward | 5’-GTG TTT TAT TAG CAA GTA TTG TGC T-3’ | 1,515 bp ^b^ |  |
|  | Reverse | 5’-CCC TAA TAA TCT CAT CCG CAG TAC G-3’ |  |  |
| 18S rRNA | Forward (609F) | 5’-CAC CCG CGG TAA TTC CAG C-3’ | 842 bp ^c^ | Da Silva *et al.,* 2004 (17) |
|  | Reverse (706R) | 5’-CTG AGA CTG TAA CCT CAA -3’ |  |  |
| ITS | Forward (IR1) | 5’-GCT GTA GGT GAA CCT GCA GCA GCT GGA TCA TT-3’ | 1,157 bp ^c^ |  |
|  | Reverse (IR2) | 5’-GCG GGT AGT CCT GCC AAA CAC TCA GGT CTG-3’ |  |  |
| ITS1 | Forward (Kin2) | 5′-CGC CCG AAA GTT CAC C-3’ | 540 bp ^d^ | Desquesnes *et al.*, 2001 (12) |
|  | Reverse (Kin1) | 5′-GCG TTC AAA GAT TGG GCA AT-3′ |  |  |

^a^ NAD7, NADH-dehydrogenase subunit 7; Cox2, Cytochrome oxidase subunit 2; A6, ATPase subunit 6; 12S rRNA, 12S ribosomal RNA; ND7-CyB, NADH-dehydrogenase subunit 7-cytochromeB; MURF-ND1, Maxicircle unknown reading frame-NADH dehydrogenase subunit 1; MURF2-Cox1, Maxicircle unknown reading frame 2-cytochrome oxidase subunit 1; ND4-ND5, NADH-dehydrogenase subunits 4-5; 18S rRNA, 18S ribosomal RNA; ITS, Internal transcribed spacer and RoTat 1.2 VSG, RoTat 1.2 variable surface glycoprotein.

^b^ The PCR product sizes of maxicircle genes are predicted from *T. b. brucei* (Accession No. M94286).

^c^ The PCR product sizes of 18S rRNA and ITS region are predicted from *T. b. brucei* TREU927 (Accession No. AC012647.18).

^d^ The PCR product size of ITS1 was obtained from Desquesnes *et al.*, 2001 (12).
