# Supplementary material for: Isolation, cultivation and molecular characterization of a new Trypanosoma equiperdum strain in Mongolia
Source: Parasit Vectors. 2016 Aug 31;9(1):481. doi: 10.1186/s13071-016-1755-3 (PMC5007690; doi:10.1186/s13071-016-1755-3)
Supplement: Additional file 3: Figure S1. — The phylogenetic tree of the 18S rRNA and ITS region. A phylogenetic analysis was performed using the T. equiperdum IVM-t1, SITB818, STIB841, STIB842, BoTat1.1, T. evansi Tansui (Accession No. D89527.1), Cairo (AB551922.1), KAI.2 (AY912277), Sam.2 (AY912279.1), T. brucei TREU927 (AC012647), T. b. gambiense DAL972 (FN554966.1), T. b. gambiense Tsuua (AJ009141) and T. b. rhodesiense Utro (AJ009142) sequences. A: A phylogenetic tree based on the 18S rRNA sequence. B: A phylogenetic tree based on the ITS sequence. Figure S2. The maxicircle PCR of the Trypanozoon species. Gel electrophoresis images of the PCR products are shown in A to G, NADH-dehydrogenase subunit 7 (NAD7; 383 bp), Cytochrome oxidase subunit 2 (Cox2; 1747 bp), ATOas subunit 6 (A6; 299 bp), 12S ribosomal RNA (12S rRNA; 1597 bp in T. b. brucei GUTat3.1 strain and T. equiperdum STIB818 strain, 1415 bp in T. equiperdum STIB841, STIB842, BoTat1.1 strains, respectively), NADH-dehydrogenase subunit 7-cytochromeB (ND7-CyB; 1450 bp), Maxicircle unknown reading frame-NADH dehydrogenase subunit 1 (MURF-ND1; 1779 bp) and Maxicircle unknown reading frame 2-cytochrome oxidase subunit 1 (MURF2-Cox1; 1551 bp), respectively. M: the 100 bp and 1 kbp DNA ladders; Lanes 1 to 8 show T. b. brucei GUTat3.1, T. evansi IL3960, T. equiperdum IVM-t1, STIB818, STIB841, STIB842, BoTat1.1 strains and negative control (distilled water), respectively. (PPTX 164 kb) [file 13071_2016_1755_MOESM3_ESM.pptx]

## Slide 1
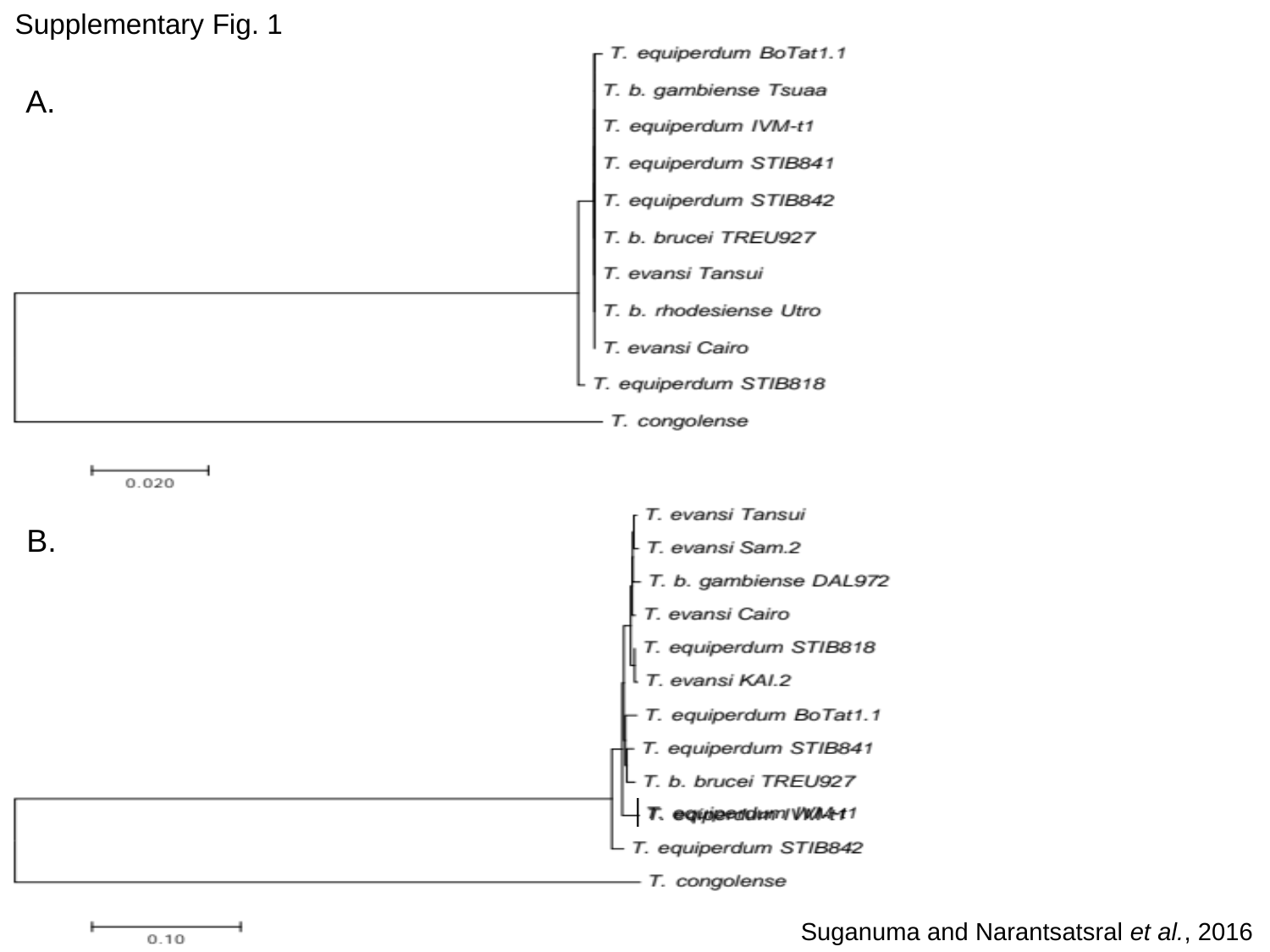

Supplementary Fig. 1
A.
B.
Suganuma and Narantsatsral et al., 2016

## Slide 2
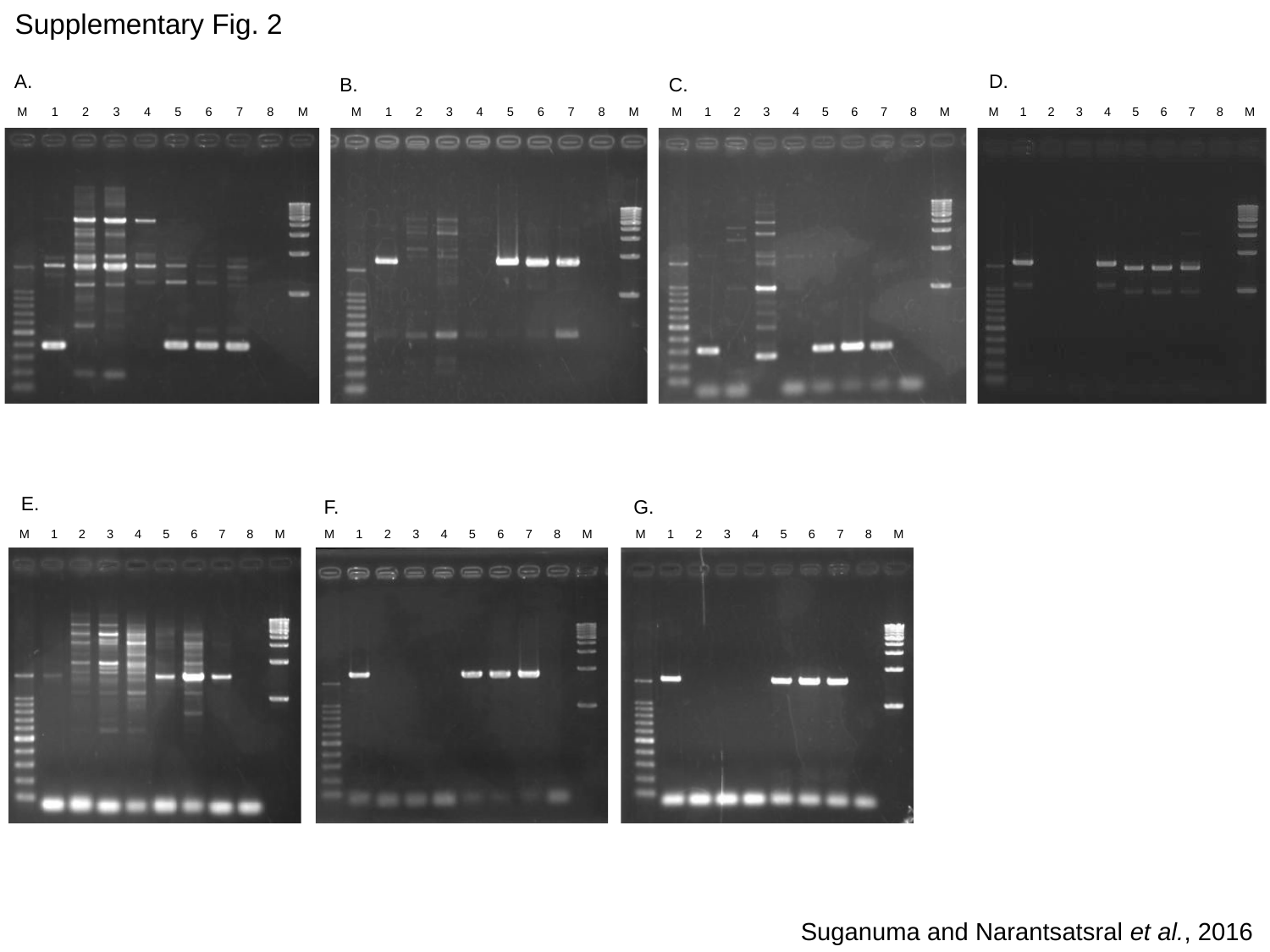

Supplementary Fig. 2
A.
D.
B.
C.
M
1
2
3
4
5
6
7
8
M
M
1
2
3
4
5
6
7
8
M
M
1
2
3
4
5
6
7
8
M
M
1
2
3
4
5
6
7
8
M
E.
F.
G.
M
1
2
3
4
5
6
7
8
M
M
1
2
3
4
5
6
7
8
M
M
1
2
3
4
5
6
7
8
M
Suganuma and Narantsatsral et al., 2016
